# Assessment of training and technical assistance needs of Colorectal Cancer Control Program Grantees in the U.S.

**DOI:** 10.1186/s12889-015-1386-1

**Published:** 2015-01-31

**Authors:** Cam Escoffery, Peggy Hannon, Annette E Maxwell, Thuy Vu, Jennifer Leeman, Andrea Dwyer, Caitlin Mason, Shaina Sowles, Ketra Rice, Lindsay Gressard

**Affiliations:** Rollins School of Public Health, Emory University, 1518 Clifton Road, 5th Floor, Atlanta, GA 30322 USA; University of Washington, Health Promotion Research Center, 1107 NE 45th Street, Ste. 200, Seattle, WA 98105 USA; University of California, 650 Charles Young Drive South (A2-125 CHS), Box 956900, Los Angeles, CA 90095-6900 USA; University of North Carolina at Chapel Hill, School of Nursing, Room 2006, Chapel Hill, NC 27599 USA; University of Colorado, 13001 East 17th Bldg 500, Office Number W6104-D, Aurora, CO 80045 USA; University of Washington, Department of Health Services, Campus Mail Box 354804, 1107 NE 45th Street, Suite 200, Seattle, WA 98105 USA; Washington University, Campus Box 1009, North Campus, Room 1549, 700 Rosedale Avenue, St. Louis, MO 63112 USA; Centers for Disease Control and Prevention, Division of Cancer Prevention and Control, 4770 Buford Highway, N.E., MS F-76, Atlanta, GA 30341 USA

**Keywords:** Colorectal neoplasms, Early detection and screening, Technical assistance, Training, Evidence-based interventions, Cancer screening

## Abstract

**Background:**

Practitioners often require training and technical assistance to build their capacity to select, adapt, and implement evidence-based interventions (EBIs). The CDC Colorectal Cancer Control Program (CRCCP) aims to promote CRC screening to increase population-level screening. This study identified the training and technical assistance (TA) needs and preferences for training related to the implementation of EBIs among CRCCP grantees.

**Methods:**

Twenty-nine CRCCP grantees completed an online survey about their screening activities, training and technical assistance in 2012. They rated desire for training on various evidence-based strategies to increase cancer screening, evidence-based competencies, and program management topics. They also reported preferences for training formats and facilitators and barriers to trainings.

**Results:**

Many CRCCP grantees expressed the need for training with regards to specific EBIs, especially system-level and provider-directed EBIs to promote CRC screening. Grantees rated these EBIs as more difficult to implement than client-oriented EBIs. Grantees also reported a moderate need for training regarding finding EBIs, assessing organizational capacity, implementing selected EBIs, and conducting process and outcome evaluations. Other desired training topics reported with higher frequency were partnership development and data collection/evaluation. Grantees preferred training formats that were interactive such as on-site trainings, webinars or expert consultants.

**Conclusions:**

Public health organizations need greater supports for adopting evidence-based interventions, working with organizational-level change, partnership development and data management. Future capacity building efforts for the adoption of EBIs should focus on systems or provider level interventions and key processes for health promotion and should be delivered in a variety of ways to assist local organizations in cancer prevention and control.

**Electronic supplementary material:**

The online version of this article (doi:10.1186/s12889-015-1386-1) contains supplementary material, which is available to authorized users.

## Background

Colorectal cancer (CRC) is the second leading cause of cancer-related deaths in the United States [1]. Routine screening makes it possible to detect and treat CRC in its early stages and thereby reduce mortality. However, in 2012, only 65% of adults were current with recommended CRC screening, with screening rates lower among the uninsured, 55% of whom had never been screened [2]. Based on extensive research testing the efficacy of interventions to increase CRC cancer screening, the *Guide to Community Preventive Services* (Community Guide) recommends the implementation of the following evidence-based interventions (EBIs): client reminders, small media, one-on-one education, provider reminders, provider assessment and feedback, and reduction of structural barriers [3-5]. Yet cancer control planners are making only limited use of these recommended EBIs [6,7]. The challenge now is to promote the use of EBIs in cancer control practice.

Disseminating information about EBIs via electronic or print media (e.g., webinars, toolkits) is widely recognized as a necessary but insufficient step towards promoting the adoption and implementation of EBIs in practice [8,9]. This is, in part, because public health and other community-based practitioners lack the knowledge and skills required to locate, adapt, and implement EBIs [9-11]. Therefore, practitioners need training and technical assistance to build their capacity to select, adapt, and implement EBIs into practice [12-14]. Training refers to instructional activities that are pre-planned and provided in group settings with the goal of increasing participants’ knowledge and skills and changing their attitudes related to EBIs. Technical assistance (TA) typically follows training and is individualized to the specific needs of individuals or staff teams [15]. Training and TA have the greatest potential to influence practice when they are tailored to practitioners’ training preferences and are designed to address gaps in their competence to perform the specific skills needed for a task [16,17].

The Interactive Systems Framework is a conceptual model that describes how organizations build capacity to use effective prevention interventions or strategies in communities [14]. Three systems work together to influence adoption of these strategies: prevention synthesis and translation (reviewing and translating research findings for practitioners), support (provision of training and technical assistance), and delivery system (implementation of effective strategies). It provides a model for understanding the facilitation and supports necessary to increase adoption of evidence-based practices in communities.

CDC launched the Colorectal Cancer Control Program (CRCCP) in 2009 in 25 states and four tribal organizations through cooperative agreements [18]. The purpose of the CRCCP is to promote CRC screening to increase population-level screening rates to 80% and, subsequently, to reduce CRC incidence and mortality [18]. The CRCCP includes two program components: 1) CRC screening of low-income, uninsured and underinsured persons and 2) implementation of EBIs aimed at increasing population-level screening rates. CRCCPs are encouraged to implement EBIs at the organizational, community, and policy levels to yield the greatest impact. To meet this goal, grantees establish partnerships with a wide range of organizations and healthcare providers in their area.

Grantees are encouraged to implement one or more of the five *Community Guide-*recommended EBIs to increase colorectal cancer screening: small media, client and provider reminders, reducing structural barriers, and provider assessment and feedback. Although these EBIs are promoted, their implementation is uneven [7]. There is a scarcity of knowledge about grantees’ training and TA needs required to implement these EBIs or the training and TA they provide to support partners’ adoption or adaptation of those EBIs. Therefore, the purpose of this study was to identify the training and TA needs and preferences for training among CRCCP grantees related to the implementation of EBIs. Assessment of training needs, preferred training approaches and barriers to participating in training and TA will inform future professional development programs for CRCCP grantees and cancer control planners in general.

## Methods

The 2012 CRCCP Grantee Survey was self-administered online in Fall 2012, using DatStat Illume™. All CRCCP program directors (PDs, N = 29) received a personalized invitation email to complete the survey. The invitation was co-signed by CDC and the Cancer Prevention and Control Research Network (CPCRN) which is conducting annual grantee surveys in partnership with CDC. The CPCRN aims to promote the translation of cancer-related evidence-based practices into local communities [19]. PDs were asked to identify the person most knowledgeable about day-to-day operations of their program’s CRCCP efforts to complete the survey. A unique link to the survey was included in each invitation. Additionally, CDC program evaluation staff sent an introduction of the survey to the PDs in advance of its fielding to alert them to the survey and encourage survey participation. The University of Washington declared the survey questionnaire and procedures exempt from IRB/human subjects review.

The questionnaire covered several topics; data presented in this paper focus on respondents’ use of the Community Guide-recommended EBIs for CRC screening promotion, barriers to use, training and TA needs on the Community Guide-recommended EBIs and EBI-related competencies, and resources used by or provided to grantees to support EBI implementation. All items were pilot-tested to assess clarity and feasibility of survey completion. The Interactive Systems Framework served as a theoretical framework for the development of questions; some focused items on general capacity building in using EBIs and others on intervention-specific items (i.e., client or provider reminders) [14]. The final questionnaire was revised based on feedback from the pilot-test.

### EBI use and desire for EBI training or TA

Grantees’ use of each of the five *Community Guide*-recommended EBIs was measured (i.e., currently use, previously used, intend to use in the next 12 months, do not intend to use in the next 12 months). For each EBI in current use, respondents were asked to rate the ease of EBI implementation on a 5-point Likert**-**like scale (1 = very difficult; 5 = very easy). Respondents were also asked to select the evidence-based strategy/ies, if any, for which they would like additional training or TA.

### Training and TA needs on EBI competencies

Grantees self-rated their desire for additional EBI- related competencies on a three point scale (1 = low desire, 3 = high desire). Competency topics included identifying EBIs, assessing the strength of the evidence for an EBI, assessing EBI fit with their population, adapting EBIs, assessing organizational capacity to implement an EBI, and evaluating EBI implementation.

### EBI resources

Respondents were asked to specify the resources they use (open-ended), to support their implementation of *Community Guide*-recommended EBIs for CRC screening promotion. Respondents were also asked to identify any partners other CRCCP or non-CRCCP funded site with whom they collaborate to implement EBIs and to indicate whether or not they provided trainings or TA to others (e.g. contractors, partners) about the use of EBIs to increase CRC screening.

### Training preferences, barriers, and satisfaction

Respondents selected from a list of the training and TA formats they preferred (e.g., on-site, online, real-time webinar, as needed consultation). Barriers to grantees’ participation in training and TA were measured by asking respondents to select up to three of the most significant barriers to their participation in training and TA from a list. Respondents used a 5-point Likert-like scale (1 = not at all satisfied, 5 = extremely satisfied) to rate their satisfaction with CDC-provided training and TA. Respondents were given the opportunity to also provide open-ended comments on how training and TA could be improved.

### Training needs for program implementation

Respondents were asked to identify their needs for training or technical assistance for program implementation, in general, in the areas of 1) program management, 2) partnership development, 3) screening provision, and 4) data collection and evaluation. For each area, respondents selected up to three activities from a list for which they/their staff would like additional training or TA to support their CRCCP in the coming year.

### Data analyses

Grantees entered data directly into DatStat Illume™ . The authors performed descriptive analyses of training and TA variables using SPSS version 21. Written text in partial close-ended questions were compiled and summarized.

## Results

### Respondent demographics

All 29 program grantees completed the survey. Nearly all respondents were either the CRCCP program director (52%) or the program manager (45%), or held both titles (3%). Eighty-three percent had been involved with their CRCCP for at least 12 months while 45% had been involved for three years or more. The majority (62%) had been working in the field of cancer control for more than five years.

Information about the CRCCP grantees can be found in the Additional file 1.

### EBI-related training and TA needs

The majority of grantees reported using small media (97%), client/patient reminders (76%), and reducing structural barriers (59%) (Table 1). Grantees indicated that the two client-oriented strategies were easiest to deliver: small media (M = 3.65, SD = 0.75) and patient reminders (M = 3.50, SD = 1.03). Provider reminders (M = 3.40, SD = 1.27), reducing structural barriers (M = 3.20, SD = 1.08) and provider assessment and feedback (M = 3.10, SD = 1.20) were rated as being slightly more difficult to implement. Grantees were most likely to identify these three EBIs as those for which they would like training or TA (41%-69%). They indicated that their greatest desire for training and TA (1 = low; 3 = high) was for the following competencies: identifying evidence-based strategies, assessing organizational capacity, assessing the strength of evidence to support a strategy’s effectiveness, and assessing the fit of potential strategies with their population (Table 2).

**Table 1:** **Use and ease of Evidence-Based Interventions (EBIs) and desire for training or technical assistance for implementing EBIs among CRCCP grantees**

	**Use EBI**	**Ease of EBI Implementation** ^**1**^		**Desire for Training or TA**
	N (%)	N	Mean (SD)	N (%)
Small Media	28 (97%)	26	3.65 (0.75)	2 (7%)
Patient Reminders	22 (76%)	16	3.50 (1.03)	6 (21%)
Provider Reminders	11 (38%)	10	3.40 (1.27)	12 (41%)
Reducing Structural Barriers	17 (59%)	15	3.20 (1.08)	16 (55%)
Provider Assessment and Feedback	13 (45%)	10	3.10 (1.20)	20 (69%)

^1^Based on Likert-like scale where 1 = very difficult, 5 = very easy. Only grantees currently implementing a given EBI were asked to rate ease.

**Table 2:** **Training and technical assistance needs for implementing evidence-based interventions among CRCCP grantees, N = 29**

**Desire for training** ^**1**^	**Mean**	**Median**	**SD**
Find evidence-based strategies or programs	2.32	2.00	0.71
Assess the strength of the evidence supporting program effectiveness	2.07	2.00	0.75
Assess the fit of potential strategies or programs with my population	2.03	2.00	0.73
Assess the fit of potential strategies or programs with my organization’s systems, staff, and resources	1.97	2.00	0.82
Assess organizational capacity to implement selected strategy	2.10	2.00	0.72
Adapt an evidence-based strategy or program to my population or setting	1.97	2.00	0.78
Implement a strategy/program with quality/fidelity	1.90	2.00	0.86
Conduct a process evaluation of an evidence-based strategy or program	1.86	2.00	1.86
Conduct an outcome evaluation of an evidence-based strategy	1.83	2.00	0.81

^1^Based on Likert-like scale where 1 = low, 3 = high.

Specific evidence-based interventions employed by each CRCCP grantee can be found in the Additional file 2.

### EBI-related resources

The majority of grantees (69%) have access to someone who can help them apply evidence. In terms of the grantees own provision of training and TA, 41% offer training on using EBIs and 45% offer technical assistance to others on using EBIs for colorectal cancer prevention.

### Training preferences, barriers and satisfaction

The majority of respondents reported a preference for training formats that are interactive in nature, including: on-site training/workshops (62%), real-time webinars (62%), and as-needed expert consultants (55%) (Table 3). Only a small portion of grantees preferred less interactive training formats such as self-directed learning materials (14%) and CD-ROM/DVD resources (3%).

**Table 3:** **Preferences for training and barriers to participation in training and Technical Assistance (TA) among CRCCP grantees, N = 29**

	**N**	**%**
**Preferred Approaches** ^**1**^		
On-site training/workshop	18	62%
Real time webinar with archiving for future use	18	62%
Expert consultant I can contact as needed	16	55%
Peer network/collaborative group/community of practice	12	41%
Online course	10	34%
Self-directed print learning materials	4	14%
CD-ROM/DVD training and resources	1	3%
Other	1	3%
**Barriers to participating in Training and TA**		
Program examples too different from my program to be helpful	16	55%
State travel restrictions not related to cost	12	41%
Information is typically too basic	11	38%
No time	9	31%
Information is impractical for everyday use	6	21%
Real world examples are not typically provided	7	24%
Money to cover travel costs	5	17%
Other	5	17%

^1^Participants could choose more than one response for training approaches, so percentages may sum to >100%.

Grantees’ most frequently reported barrier to participating in training was the perception that program examples described during training were too different from their own program (55%). Other frequently reported barriers included travel restrictions not related to cost (41%) and a perception that the information provided at the training is too basic (38%). Only 17% of grantees reported that travel cost issues were a barrier to participation. On average, grantees reported moderate levels of satisfaction with both CDC-provided training (M = 3.14, SD = 0.95) and technical assistance (M = 3.17, SD = 1.00).

### Training needs for program implementation

The five most frequently reported needs in each area of program implementation are summarized in Table 4. Training needs related to program management were varied; no single area of training was selected by more than one third of grantees. Comprehensive program planning was most frequently reported (31%). In the area of partnership development, grantees frequently reported a need for assistance in developing and maintaining partnerships with key stakeholders in health care, including private health insurers (55%), Medicare or Medicaid (41%), private and nonprofit health care systems (38%), federally qualified health centers (24%), and professional organizations (17%). For screening provision, grantees most frequently reported a need for assistance in providing education to health care professionals who are funded through their program (34%), facilitating insurance enrollment (28%), and securing treatment for patients diagnosed with cancer (24%). In the area of data collection and evaluation, nearly half of respondents (45%) reported a need for training in identifying and collecting data from sources other than their program’s providers. Grantees also reported a need for assistance in conducting evaluation activities (38%) and, less frequently, in developing an evaluation plan for their program (21%). Table 5 presents some capacity building resources that CDC has offered to grantees that covers these program areas.

**Table 4:** **CRCCP Grantees’ training and technical assistance needs for program implementation**

**Training topics**	**N**	**%**
**Program Management**		
Comprehensive program planning	9	31%
Integrating with other programs	6	21%
Recruiting providers for screening provision	5	17%
Communication	5	17%
Working with or managing contractors	5	17%
**Partnership Development**		
Develop and maintain partnerships with private health insurers	16	55%
Develop and maintain a relationship with your State Medicare and Medicaid office	12	41%
Develop and maintain partnerships with private and nonprofit health care systems	11	38%
Develop and maintain a partnership with FQHCs	7	24%
Develop and maintain partnerships with professional organizations	5	17%
**Screening Provision**		
Develop, promote, or enhance training to educate health care professionals among program-funded providers	10	34%
Support insurance enrollment	8	28%
Ensure appropriate treatment for complications and cancers	7	24%
Develop and promote quality control standards and mechanisms among program-funded providers	7	24%
Convene and maintain a Community Advisory Board	6	21%
**Data Collection and Evaluation**		
Identify and collect data from other sources (e.g., CRC screening rates from large health systems)	13	45%
Conduct evaluation activities for your CRC efforts	11	38%
Implement strategies to document and communicate program value to stakeholders (e.g. legislators, funders, administrators)	9	31%
Use data for program monitoring and program improvement	7	24%
Develop an evaluation plan for your CRC efforts (e.g., formative, process, outcome, impact)	6	21%

**Table 5:** **CDC provided training and technical assistance activities to All CRCCP grantees during program years 2012-2013**

**CDC-Provided training and technical assistance activities**	**Training areas/Categories**	**Type of training**
Collaboration Across CDC Cancer Programs	Partnership Development	Webinar
QSST Presentation: Using CCDEs to Assess Screening Quality	Data Management	Webinar
Improving Implementation of Evidence-based Interventions: The Example of Small Media	EBI Implementation	Webinar
Academic Detailing	Professional Development & Provider Education	Webinar
Using Logic Models as Tools for Planning and Evaluation	Program Planning	Webinar
Improving Cancer Screening Outcomes in Rural Areas	Program Outreach	Webinar
Cost Assessment Tool Training	Data Management	Webinar
Systems Change via Provider Feedback	Systems Change/EBI Implementation	Webinar
Community Health Workers: Examples from the Field	Public Education & Targeted Outreach	Webinar
Key Considerations in Designing a Navigation Program	Patient Navigation	Webinar
Population-Based and Systems Change Activities	Systems Change/EBI Implementation	Webinar
Using Your PETO Logic Model for Program Planning	Program Planning	Webinar
AMIGAS Project: Bilingual Education Outreach Intervention	Program Outreach	Webinar
Systems and Policy Change Training	Systems Change	On-site Training
Seizing Opportunities Provided by Expanded Clinical Preventive Services	Public Education & Targeted Outreach	Webinar

## Discussion

A large proportion of CRCCP grantees expressed the need for training with regards to specific EBIs, especially system-level EBIs and provider-directed EBIs to promote CRC screening. Grantees rated these EBIs as more difficult to implement than client-oriented EBIs and fewer grantees reported use of these EBIs. Grantees also reported a moderate need for training regarding many aspects of EBI implementation, from identifying EBIs and assessing organizational capacity to implement selected EBIs to conducting a process and outcome evaluation. CDC has provided webinars to grantees on many of these topics, but the moderate satisfaction rating of grantees with CDC training and TA suggests that there is room for improvement. CDC may benefit from gathering specific information from grantees on how to improve their training and TA efforts. Meeting grantees’ training and TA needs is important, as their capacity to implement EBIs will be crucial for meeting the program goal of increasing levels of CRC screening population-wide. Generally, the CRCCP grantees were using EBIs to increase colorectal cancer screening recommended by the CDC; however, they desired training and TA on the specific interventions that require organizational or systems changes, such as provider assessment and feedback and reducing structural barriers. Research-tested Intervention Programs (R.T.I.P.s) may be a useful resource for grantees to locate specific interventions with their implementation protocols and materials [20]. To assist public health organizations in correctly implementing EBIs, it is critical to offer intervention-specific guidance in addition to general instructions on how to use evidence as suggested by the Interactive Systems Framework [14]. For EBIs that demand changes to organizational procedures or processes, support of administrators is often needed. Toolkits, such as the American Cancer Society’s *How to Increase Colorectal Cancer Screening Rates in Practice* [21] may be useful in guiding organizations and clinics to engage relevant administrators and adopt those provider-oriented evidence-based strategies [14]. Further training and TA on these provider-oriented strategies are needed.

For general capacity building, we found that grantees desired training and TA on specific topics regarding EBIs such as identifying evidence-based strategies, assessing organizational capacity, adapting EBIs, and assessing the fit of potential strategies with their population. This is consistent with findings of previous research assessing training needs to increase the use of EBIs among other health professionals [22]. CDC’s Comprehensive Cancer Program staff has also reported topics of adapting EBIs and maintaining fidelity as desired training areas [11]. And, among health department staff, translating EBIs and evaluation competencies were rated as intermediate or advance competencies for training [23]. Several models of adoption of effective interventions emphasize the need for conducting a fit assessment to determine if an evidence-based program, policy, or practice matches the community’s capacity, resources, or readiness to act in order to implement an intervention correctly [24,25]. Likewise, organizational capacity checklists exist that could help organizations assess their resources and readiness to implement an EBI [26-28]. Training for public health professionals can focus on these content areas to assist organizations with these specific tasks within using EBIs. Our results also suggest that CRCCP grantees are active disseminators of EBIs in that 40-50% of them offer training and TA to other organizations. These grantees represent active prevention support to others in terms of sharing their how-to’s of EBI implementation, facilitators and barriers, and lessons learned.

Many grantees preferred training formats that were interactive with content experts such as onsite training, webinars or expert consultants around the implementation of systems approaches for increasing screening; however, resource constraints often limit CDC staff from offering onsite or in-person training. While training is necessary, individualized TA may be more suitable to help organizations overcome the training barriers identified in this study (e.g., need for examples, more advanced information). Individualized TA can provide more in-depth information on how to implement a specific strategy and also address unique contextual factors (e.g., organizational systems such as paper records or electronic medical records, staffing, resources). These combinations of prevention support reported by grantees match the common strategies found in other studies that promote the adoption of EBIs such as in person training and TA [29,30], packaged materials or recommendations [31,32], and conference calls [33]. These prevention supports have been proven to increase adoptions of evidence-based prevention strategies [31,32,34].

In addition to systems approaches for increasing screening, the needs for training and TA around partnership development and program and data management were most frequently reported by grantees as critical training topics. Because there are still disparities in cancer screening, building grantees’ and other community organizations’ capacity to leverage or build partnerships such as those between clinical and community preventive services would promote screening [35]. In qualitative interviews with over 100 stakeholders from the Colorectal Cancer Screening Demonstration Program, data revealed that partnership development and collaborations were critical to the success of the demonstration sites [36].

These findings present some practical implications for CDC and other organizations supporting the use of EBIs. The diversity inherent in the aforementioned categories of training needs and preferences can be challenging for a systematically designed program such as the CRCCP. Recognizing this potential challenge, CDC proactively implements a four-pronged effort for training and TA which 1) establishes a Program Consultant for each grantee to provide in-person and/or telephonic individual, tailored TA on an ongoing basis, 2) establishes training and TA workgroups on specific topics (e.g., education and targeted outreach, quality assurance and patient navigation) that offer webinars and in-person training, 3) supports development of training or TA materials (e.g., action guides such as *Increasing Quality Colorectal Cancer Screening: An Action Guide for Working with Health Systems* [37], Make It Your Own [MIYO] [38], and 4) provides in-person training at conferences. These efforts aim to provide systematic, universal training as part of the overall capacity building effort for all CRCCP grantees.

Program Consultants provide individual TA and consultation to assigned grantees to support their planning, implementation and evaluation of each of their program components. TA and consultation includes individual assistance with the design and implementation of grantee program components and data monitoring and feedback to support quality assurance, program improvement, and program evaluation. These activities represent the types of support strategies recommended by the Evidence-based System for Innovation Support to increase adoption of EBIs: training, TA, tools and quality improvement [15]. Universal training provided by CDC during the 2012–2013 program year included a range of activities in response to grantee’s requests from the 2012 survey (Table 5) and involved real-time webinars and on-site training for expanding grantee’s capacity in systems approaches/EBI implementation, program management, data management, and partnership development. In addition, the webinars and on-site training provided by CDC often include presentations developed by grantees in order to highlight their successes with EBI implementation. These examples from the field have proven useful for grantees still having difficulty with program implementation and encourage collaboration and knowledge sharing among grantees.

CDC’s approach of establishing a Program Consultant for individualized TA and an education and targeted outreach team for universal training allows for greater responsiveness to the needs of grantees and also encourages grantees to continually keep the agency informed of their challenges and training desires. This level of communicative feedback is integral so that continual improvement can be made in overall CRCCP program outreach and management. In addition, because CDC instituted a performance-based grants management system in 2012 for annual funding of CRCCP grantees, CDC is able to integrate training and TA efforts with evaluation of grantee performance. CDC is then able to follow up with programs in need of assistance to increase their capacity.

This study has several limitations. Our findings are based on self-reported data from grantees who are funded for promoting CRC screening at the population level using EBIs and that have received a significant amount of training related to program implementation in general and related to specific EBIs. It is possible that training and TA needs are different, possibly greater, for other organizations or clinics that focus on colorectal screening promotion. In addition, we limited the focus of the desire for training and TA to currently recommended evidence-based practices. Grantees may also need guidance on other cancer promotion activities such as mass media campaigns, reducing screening costs, and group education, where the evidence has not been established or is insufficient.

Future research can explore the outcomes of training and TA on short-term changes such as knowledge and skills gained on how to implement EBIs. Further long-term evaluation can examine the adoption of recommended cancer prevention EBIs by public health organizations and subsequent impact on screening. While general training on the use of EBIs exists [16,39], few evaluations are published that present critical data in terms of what knowledge and competency areas are important to address for dissemination of evidence-based practices for cancer control.

## Conclusion

The goal of the CRCCP and other public health organizations is to increase colorectal cancer screening prevalence to 80% by 2018 [18,40] and subsequently reduce colorectal cancer incidence and mortality by promoting population-level colorectal cancer screening and providing screening to uninsured, low-income individuals ages 50–64. Although there is fairly moderate use of EBIs among the grantees, greater adoption and quality implementation of these strategies may be realized through training and individualized TA provided by CDC, other cancer-focused national organizations, and cancer experts. The use of tailored and systematic, universal methods of training and TA will provide the level of assistance and support necessary to enhance grantees, and other community organizations’ capacities to ultimately reach this goal.

## Additional files

Additional file 1:
**Grantee Characteristics.**
Additional file 2:
**Use of Evidence-Based Interventions, by Grantee, 2012-2013.**
