# Supplementary material for: Assessment of training and technical assistance needs of Colorectal Cancer Control Program Grantees in the U.S
Source: BMC Public Health. 2015 Jan 31;15:49. doi: 10.1186/s12889-015-1386-1 (PMC4318175; doi:10.1186/s12889-015-1386-1)
Supplement: Additional file 1: — Grantee Characteristics. [file 12889_2015_1386_MOESM1_ESM.docx]

Table S1. Grantee Characteristics

| **Grantee** | **Primary Screening Test** | **Prioritized Populations for Screening** |
| --- | --- | --- |
| Alabama | FIT | No population subgroups are prioritized for screening |
| Alaska Native Tribal Health Consortium (ANTHC) | Colonoscopy | American Indian or Alaskan Native |
| Arizona | FIT | Black or African-American, American Indian or Alaskan Native, Hispanic, Latino or Spanish origin, Persons in specific geographic areas, Rarely or never screened populations |
| Artic Slope Native Association (ASNA) | Colonoscopy | American Indian or Alaskan Native |
| California | FIT | White, Asian, Persons in specific geographic areas, Rarely or never screened populations, Urban populations, Immigrant/refugee populations |
| Colorado | FIT | Persons in specific geographic areas |
| Connecticut | Colonoscopy | Black or African-American, Rarely or never screened populations, Urban populations |
| Delaware | Colonoscopy | Black or African-American, Rarely or never screened populations |
| Florida | FIT | Black or African-American, Hispanic, Latino or Spanish origin, Rarely or never screened populations, Haitian Creole |
| Georgia | Colonoscopy | Rarely or never screened populations |
| Iowa | FIT | We do not prioritize any population subgroups for screening. |
| Massachusetts | Colonoscopy | Rarely or never screened populations |
| Maryland | Colonoscopy | Persons in specific geographic areas, Urban populations |
| Maine | Colonoscopy | No population subgroups are prioritized for screening |
| Michigan | FOBT | Black or African-American, White, Hispanic, Latino or Spanish origin, Persons in specific geographic areas, Rarely or never screened populations, Rural populations |
| Minnesota | Colonoscopy | Black or African-American, American Indian or Alaskan Native, Rarely or never screened populations, Rural populations, Urban populations, Men |
| Montana | Colonoscopy | American Indian or Alaskan Native, Rural populations |
| Nebraska | FOBT | Black or African-American, White, Asian, American Indian or Alaskan Native, Native Hawaiian or other Pacific Islander, Hispanic, Latino or Spanish origin, Persons in specific geographic areas, Rarely or never screened populations, Rural populations, Urban populations |
| New Hampshire | Colonoscopy | Persons in specific geographic areas, Rural populations, Urban populations, Immigrant/refugee populations, Low socioeconomic and individuals with high school or less education |
| New Mexico | FIT | White, American Indian or Alaskan Native, Hispanic, Latino or Spanish origin, Persons in specific geographic areas, Rarely or never screened populations, Rural populations, Those populations in which Social Determinants of Health need to be addressed |
| Nevada | FIT | Rarely or never screened populations |
| New York | Colonoscopy | Black or African-American, Hispanic, Latino or Spanish origin, Persons in specific geographic areas, Rarely or never screened populations, Urban populations, Immigrant/refugee populations |
| Oregon | FIT | No population subgroups are prioritized for screening |
| Pennsylvania | Colonoscopy | Black or African-American, Asian, Rarely or never screened populations, Urban populations, Immigrant/refugee populations |
| South Dakota | FIT | White, American Indian or Alaskan Native, Hispanic, Latino or Spanish origin, Persons in specific geographic areas, Rarely or never screened populations, Rural populations, Immigrant/refugee populations |
| Southcentral Foundation | Sigmoidoscopy | American Indian or Alaskan Native |
| South Puget Intertribal Planning Agency (SPIPA) | FIT | American Indian or Alaskan Native |
| Utah | Colonoscopy | 200% federal poverty, first time screened and asymptomic, no insurance, 50-64 years, legal US and Utah citizen, average risk and referred by PCP (primary care provider) |
| Washington | FIT | Black or African-American, Asian, American Indian or Alaskan Native, Hispanic, Latino or Spanish origin, LGBTQ |
