# Supplementary material for: Assessment of training and technical assistance needs of Colorectal Cancer Control Program Grantees in the U.S
Source: BMC Public Health. 2015 Jan 31;15:49. doi: 10.1186/s12889-015-1386-1 (PMC4318175; doi:10.1186/s12889-015-1386-1)
Supplement: Additional file 2: — Use of Evidence-Based Interventions, by Grantee, 2012-2013. [file 12889_2015_1386_MOESM2_ESM.docx]

Table S2. Use of Evidence-Based Interventions, by Grantee, 2012-2013

|  |  | **Small Media** | **Client Reminders** | **Provider Reminders** | **Provider Assessment & Feedback** | **Reducing Structural Barriers** | **Total EBIs per grantee** |
| --- | --- | --- | --- | --- | --- | --- | --- |
| AC | ASNA | - | - | - | - | - | **-** |
| AL | Alabama | Yes | Yes | Yes | Yes | Yes | **5** |
| AN | ANTHC | Yes | Yes | Yes |  | Yes | **4** |
| AZ | Arizona | Yes | Yes | Yes | Yes | Yes | **5** |
| CA | California | Yes | Yes | Yes | Yes | Yes | **5** |
| CO | Colorado | Yes | Yes | Yes | Yes | Yes | **5** |
| CT | Connecticut | Yes |  |  |  | Yes | **2** |
| DE | Delaware | Yes | Yes | Yes |  |  | **3** |
| FL | Florida | Yes | Yes | Yes |  |  | **3** |
| GA | Georgia | Yes | Yes | Yes | Yes | Yes | **5** |
| IA | Iowa | Yes | Yes |  |  | Yes | **3** |
| MA | Massachusetts | Yes | Yes | Yes | Yes | Yes | **5** |
| MD | Maryland | Yes | Yes | Yes | Yes | Yes | **5** |
| ME | Maine | Yes |  |  |  | Yes | **2** |
| MI | Michigan | Yes | Yes | Yes | Yes | Yes | **5** |
| MN | Minnesota | Yes | Yes |  |  | Yes | **3** |
| MT | Montana | Yes | Yes | Yes | Yes | Yes | **5** |
| NE | Nebraska | Yes | Yes |  |  |  | **2** |
| NH | New Hampshire | Yes | Yes | Yes | Yes | Yes | **5** |
| NM | New Mexico | Yes | Yes | Yes | Yes | Yes | **5** |
| NV | Nevada | Yes | Yes | Yes |  | Yes | **4** |
| NY | New York | Yes |  | Yes | Yes | Yes | **4** |
| OR | Oregon | Yes |  |  |  |  | **1** |
| PA | Pennsylvania | Yes |  | Yes | Yes | Yes | **4** |
| SD | South Dakota | Yes | Yes |  |  | Yes | **3** |
| SO | Southcentral | Yes | Yes |  |  |  | **2** |
| SP | SPIPA | Yes | Yes | Yes |  | Yes | **4** |
| UT | Utah | Yes | Yes | Yes | Yes | Yes | **5** |
| WA | Washington | Yes | Yes |  | Yes | Yes | **4** |
|  | | **28** | **23** | **19** | **15** | **23** |  |
